# A Study of the Thermal Degradation and Combustion Characteristics of Some Materials Commonly Used in the Construction Sector

**DOI:** 10.3390/polym11111833

**Published:** 2019-11-07

**Authors:** Javier Arturo Piedrahita Solorzano, Khalid Abu Mohammad Moinuddin, Svetlana Tretsiakova-McNally, Paul Joseph

**Affiliations:** 1 Institute of Sustainable Industries and Liveable Cities, Victoria University, PO Box 14428, Melbourne, VIC 8001, Australia; piedramaths@gmail.com (J.A.P.S.); Khalid.Moinuddin@vu.edu.au (K.A.M.M.); 2 Belfast School of Architecture and the Built Environment, Ulster University, Newtownabbey BT37 0QB, Northern Ireland, UK; s.tretsiakova-mcnally@ulster.ac.uk

**Keywords:** façade materials, thermal degradation, combustion characteristics, correlations

## Abstract

In the present work, some materials that are commonly used in the construction industry were studied with regard to their thermal degradation characteristics and combustion attributes. These included façade materials for pre-fabricated houses, such as the layers of cross-laminated timber (CLT) and the inner core of aluminium composite panels (ACPs). The relevant investigations were carried out by employing thermo-gravimetric analysis (TGA) and pyrolysis combustion flow calorimetry (PCFC). The Arrhenius parameters and the associated calorimetric quantities, i.e., heat release rates, temperature to the peak heat release rate, heats of combustion, heat release capacities, and char yields, were also evaluated. These parameters showed that CLT is more fire retarded than the polymeric internal core of ACP façade materials. Furthermore, some valuable correlations among the various test quantities were found. For instance, a good correlation exists between the general profiles of the thermograms obtained through TGA runs and the heat release rate (HRR) traces from PCFC measurements. Depending on the nature of the materials, the char yields measured by PCFC can be 4–20 times higher than the ones obtained through TGA.

## 1. Introduction

Generally, the assessment of fire risks for the new materials involves various approaches for collating different fire-related parameters [1,2]. They include, but not limited to, the radiant heat flux generated, the amounts of smoke and toxic gaseous components produced (e.g., carbon monoxide CO and carbon dioxide CO_2_), the fire spread rate and the propensity of construction elements to result in structural failures. The associated material’s properties of interest are times (or temperatures) to ignition; heat release rates (HRRs); heat release capacities (HRCs); char residues; and kinetic parameters pertaining to the thermal degradation [3]. In order to measure the experimental quantities of interest, a variety of small-, medium-, and large-scale tests are usually performed. These include: A corner room test ISO 9705 (large-scale) [4]), cone calorimetry (medium-scale) [5]), and small-scale tests such as thermo-gravimetric analysis (TGA) [6] and pyrolysis combustion flow calorimetry (PCFC) [7]. 

There have been several attempts to find correlations between the empirical data obtained through small- and medium-scale tests [8] with a view to predicting an overall reaction of materials to real-life fires. For instance, Östman and Nussbaum found an empirical correlation between the times to flashover in a full-scale room fire test for surface lining materials from the measurements of the rate of heat release in a cone calorimeter [9]. Furthermore, cone calorimetry results were employed by Wickström and Göransson to predict the heat release rate of surface materials in a large-scale fire test [10]. Dowling and Feske estimated the fire spread and smoke evolution of flame-retarded rigid polyurethane (FR-PUR) from the relevant cone data [11]. 

However, not all of these attempts were fully successful. For example, Weil et al. (1992) attempted, with a limited success, to correlate the cone calorimetry measurements and the limiting oxygen indices (LOIs) [12]. Similarly, Hirschler had found only partial correlations among various data obtained through studies of the HRRs for twenty-one types of electrical cables, using several small-scale fire tests (e.g., various small burner-type and cone calorimeter tests), three European medium-scale cable tray tests (DIN 4102, Brandschacht; BS 476/Part 12 Ignition source E; IEC 1034), and two large-scale vertical cable tray tests (ASTM D5424#5537 (CSA FT4) and IEC 332-3) [13].

The correlation studies between the results from TGA (both in air and in nitrogen) of nine different ‘flame-resistant’ and one ‘non-flame resistant’ rubber conveyor belts, LOI measurements, hot plate ignition, and drum friction tests (i.e., other flammability tests) were also reported in the literature [14]. Generally, the relevant empirical parameters such as activation energy and pre-exponential factor are very useful in validating some existing mathematical/computational models pertaining to the ignition characteristics and fire behaviours of the materials. For instance, these kinetic parameters can be used for combustion and pyrolysis sub-models of a computational model, simulating the fire spread on a defined woodland [3].

Through the current research, we undertook various experimental testing to gain insights into the ignition behaviour and combustion characteristics of two materials that are commonly used in the construction sector: Cross-laminated timber (CLT) and the inner core of aluminium composite panels (ACPs). In recent years, the chosen materials were severely implicated in the initiation, or the spread of fires, in enclosures and exteriors of the buildings [1,2]. For example, ACPs for external façades caught the attention of the scientific community following the Grenfell Tower fire in London [1]. It is believed that a flammable polyethylene-based core of ACPs has contributed to a rapid fire spread outside the tower block. Other examples of fires with similar materials involved happened in the Middle East, China, and Australia. The overall aim of the present work is therefore to obtain and analyse the relevant information pertaining to the fire-safe use of CLT and ACPs in the built environment. In order to achieve this aim, we have employed some of the relevant analytical techniques to gather the required data. Here, we have also endeavoured to seek analytical correlations, if any, between the experimental results, with a view to gauging the materials’ behaviours in real fire scenarios.

## 2. Materials and Methods 

### 2.1. Materials

Two different classes of materials commonly used in the construction sector, the details of which are given in Table 1, were sourced locally in Melbourne, Australia. The aluminium composite panels were representative of commercially available varieties that are also sold at other parts of the world, where as the cross-laminated timber samples are unique and were produced at the Centre for Advance Manufacturing of Prefabricated Housing at the University of Melbourne, Australia. The chemical compositions of the materials were not known with certainty owing to the commercial sensitivity. However, we were able to gather some limited information regarding the chemical nature of the tested specimens from the manufactures, which revealed that the CLT specimen was predominantly ligno-cellulosic in nature, whereas the internal core material of the ACPs consisted mainly of polyethylene. Here it is relevant to note that polyethylene core ACP-type 1 material was grey in colour, whereas the corresponding material in ACP-type 2 was black in colour. While in both cases this indicates the presence of some sort of additive(s), the exact chemical nature of the additive(s) was not made available by the manufacturers. 

### 2.2. Methods

#### 2.2.1. Thermo-Gravimetric Analysis (TGA)

TGA runs were done using a Mettler Toledo instrument, at different heating rates (HRs) (5, 10, 15, 20 and 25 °C∙min^−1^), in the temperature interval from 30 to 900 °C, in the atmosphere of nitrogen, in triplicates. The underlying degradation processes were assumed to follow an Arrhenius-type kinetics according to the following relation [6]:

(1) $$\left(\frac{d\alpha }{dt}\right)=Aexp\left(-\frac{E_{a}}{RT}\right){\left(1-\alpha \right)}^{n}$$

where α—the degree of conversion (dimensionless); *t*—(s); *A*—pre-exponential Arrhenius factor (s^−1^); *E_a_*—activation energy (J∙mol^−1^); *R*—the universal gas constant (8.314 J∙mol^−1^∙K^−1^); and *n*—the reaction order.

**Figure 1:**
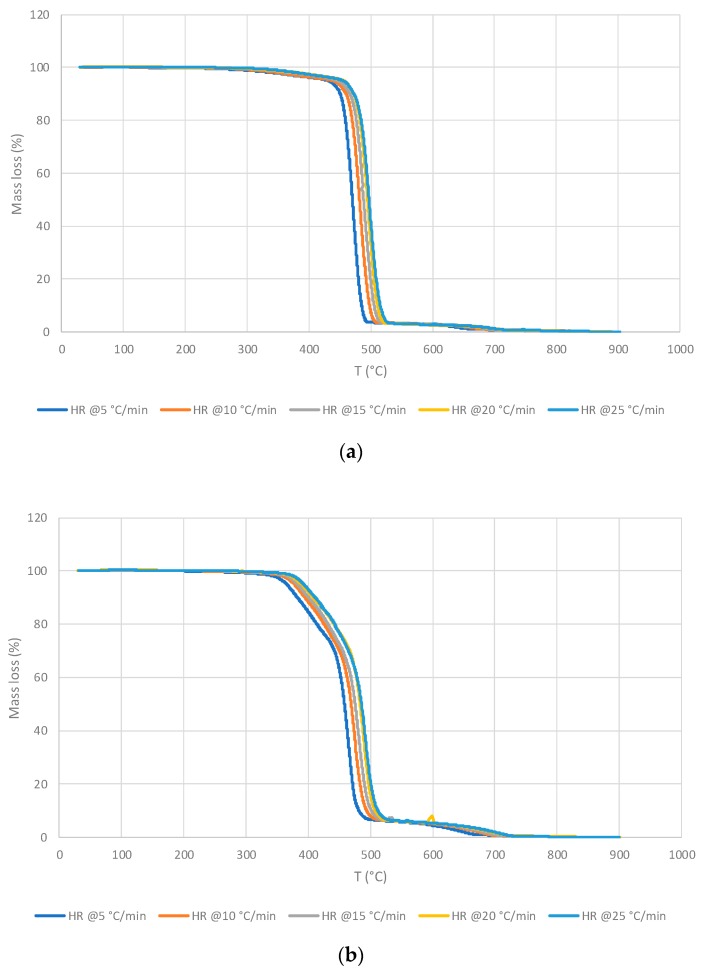
Thermo-gravimetric analysis (TGA) traces of: (**a**) Façade type 1 material; (**b**) Façade type 2 material at different heating rates (HRs) in nitrogen.

**Figure 2:**
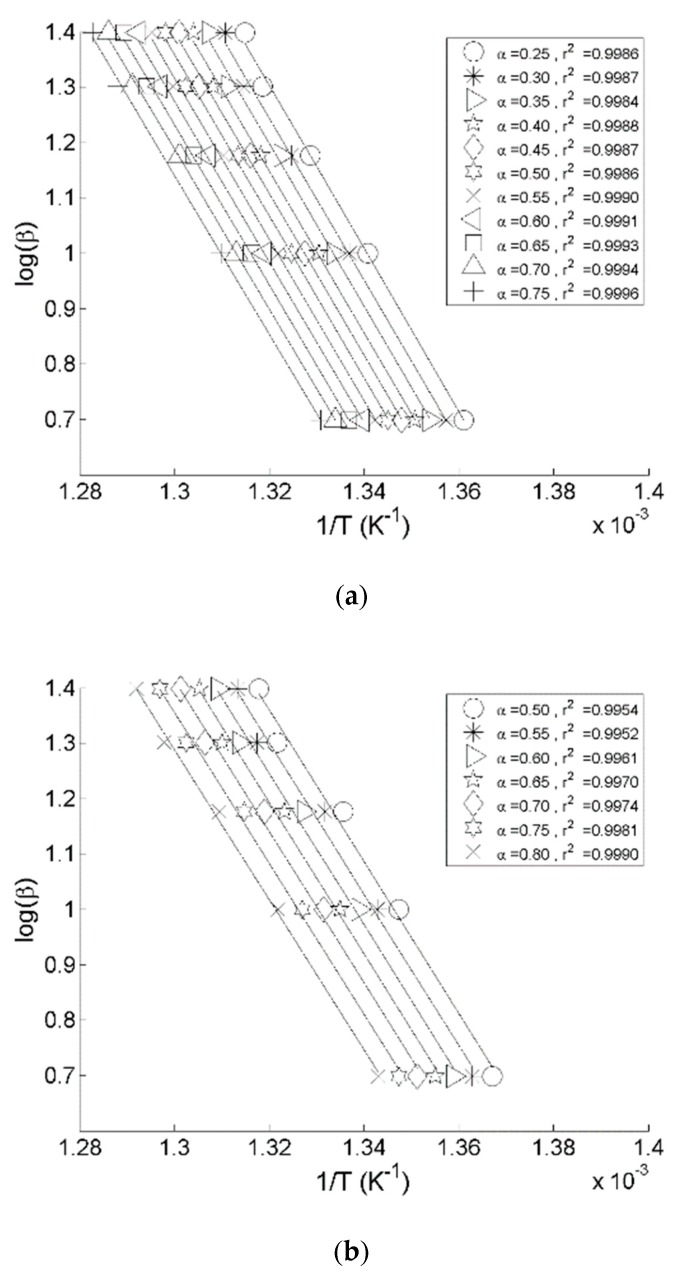
Plots logβ versus 1/T for: (**a**) Façade type 1 material; (**b**) Façade type 2 material.

According to the Flynn-Wall-Ozawa model [15], the Equation (1) was rearranged in the following way:

(2) $$E_{a}=\left(-\frac{R}{b}\right)\frac{d\ln\beta }{d\left(\frac{1}{T}\right)}=\left(-\frac{R}{b}\right)Constant$$

where *b*—the constant, assuming *n = 1;* β—the heating rate (°C∙min^−1^); and T—temperature of a weight loss (°C). Here, it is possible to calculate the Arrhenius factor (*A*) and the energy of activation (*E_a_*) through a construction of the plots logβ versus 1/T. The thermograms shown in Figure 1 were analysed to find these kinetic parameters using a code in MATLAB software [3] developed in our laboratories. In the present study, the code was programmed in conformance with the Flynn-Wall-Ozawa method to find the values of *E_a_*, *A,* and α [16]. Figure 2 illustrates typical plots of logβ versus 1/T for varying values of α that enabled calculation of the Arrhenius parameters of the studied materials. The above procedure was followed to find out the Arrhenius parameters for all the materials studied in the present work.

#### 2.2.2. Pyrolysis Combustion Flow Calorimetry (PCFC)

PCFC is a very useful technique to study the degradation characteristics and combustion attributes of solid fuels such as temperature to peak heat release rate (tPHRR), total heat release (THR), peak to heat release rate (pHRR), and heat release capacity [8]. The HRC value is a good indicator of a material’s capacity to generate heat for each degree of the temperature upon combustion, or pyrolysis [17]. The PCFC equipment allows to run experiments utilising two methods, method A and method B. Method A carries out the measurement in an inert atmosphere (i.e., in nitrogen), whereas method B uses an oxidative environment (i.e., air) [18]. It is highly relevant to note here that the THR values might significantly different which predominantly depends on the chemical nature of the substrate in question. In the present study such a difference was noted in the case of the three layers of the CLT material. For PCFC runs, a fire testing technology instrument was employed, and the samples were analysed according to both methods [7,17,18,19]. 

The apparent heat of combustion, ($h_{c}$), was calculated as per Equation (3) following a protocol detailed in [17].

(3) $$h_{c}=\frac{THR}{1-Y_{p}}$$

where *THR* is a specific heat release of the sample, i.e., the area under the corresponding HRR curve (kJ∙g^−1^); $Y_{p}$—pyrolysis residue (g∙g^−1^). For example, for a sample exhibiting THR of 18.07 kJ∙g^−1^ and leaving a 45 wt.% char residue the apparent heat of combustion was calculated as follows:

(4) $$h_{c}=\frac{THR}{1-Y_{p}}=\frac{18.07}{1-0.45}=32.86\;kJ\cdot g^{-1}$$

## 3. Results and Discussion

### 3.1. Polymeric core of the ACP 

#### 3.1.1. Thermo-gravimetric Analyses

Generally, for the inner core of the façade materials (for both façade types), the main degradation happens in one step, and essentially, is in the same temperature range regardless of the heating rate. Indeed, for the Façade type 1 material this occurred between ca. 280 and 540 °C and for the Façade type 2—between ca. 350 and 520 °C, with a second stage taking place from ca. 520 to 700 °C (Figure 1). 

It was noticed that all thermograms are very strikingly similar regardless of the HRs. The Façade type 1 material reached the maximum value for the mass loss at 484.0 °C, and left 2.8 wt.% residue at 600 °C. For the Façade type 2 material, the corresponding values were 479.0 °C and with 5.0 wt.% char yield at 600 °C. The values of kinetic parameters obtained for the façade materials are as follows: Façade type 1 has an *E_a_* of 264.55 kJ∙mol^−1^, primarily averaged between α values from 0.25 to 0.75; Façade type 2 has an *E_a_* of 249.36 kJ∙mol^−1^ with α values averaged over the range from 0.50 to 0.80 (Figure 2). It is to be noted that for both materials the respective *E_a_* values fluctuated greatly outside the abovementioned α ranges, and that within the selected α ranges the *E_a_* values turned out to be somewhat similar. However, the values of the pre-exponential factor, *A*, were lower for the Façade type 2 material by the factor of 10.

#### 3.1.2. PCFC Studies 

For the PCFC tests, the powdered samples of Façade types 1 and 2 materials (*ca.* 5–10 mg) were accurately weighed into ca. 70 cm^3^ ceramic crucibles. Both method A and method B were employed, with a HR of 25 °C∙min^−1^ (i.e., 0.416 °C∙s^−1^) until 600 °C for the façade materials [17]. The HR of 0.416°C∙s^−1^ was chosen to match it closely with the HR employed in the TGA experiments, i.e., 25 °C∙min^−1^.

The results of PCFC for the tested samples are listed in Table 2 and Table 3. As expected, the HRC and pHRR values were found to be higher in the oxidative atmosphere; however, the corresponding values for the char yield, THR, the heat of combustion, *h_c_*, and temperatures to pHRR, $T_{max}^{*}$, were similar in both atmospheres. Out of the two materials, Façade type 2 gave the best result in terms of the char yield (around 60 wt.%), which indicates its higher resistance to combustion compared to Façade type 1. This was also confirmed by the lower values of THR, pHRR, and HRC for the Façade type 2 material. The Façade 2 material is more amenable to thermal degradation as indicated by relatively lower *E_a_* value as compared to Facade type 1 material. The slightly earlier induction period coupled with an increased propensity for degradation could lead to an enhanced combustion inhibition, predominantly operating in the condensed phase for Façade type 2 material, as was also revealed through substantially higher amounts of char residues (see in Table 2 and Table 3).

**Figure 3:**
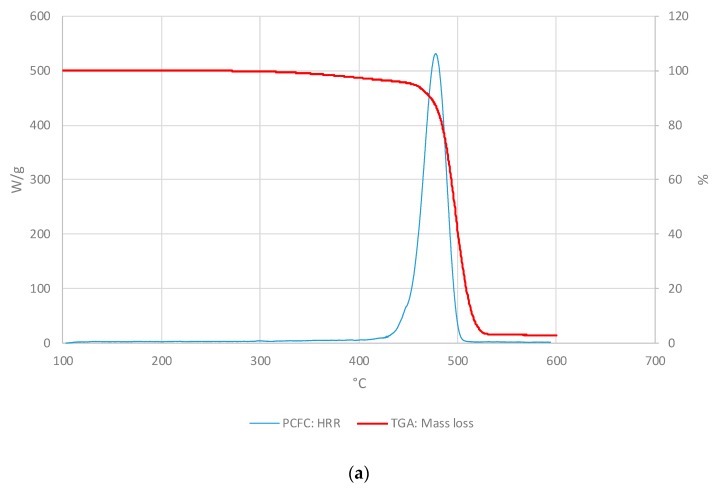

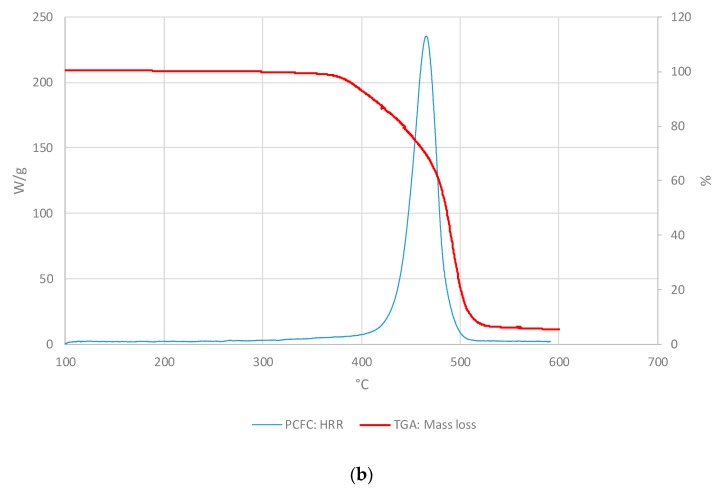
The heat release rate (HRR) (W/g) and mass loss (wt.%) curves for: (**a**) Façade type 1 material; (**b**) Façade type 2 material.

In the Figure 3, the PCFC and TGA curves were compared as the HR and the atmosphere were the same for both methods (i.e., at 25° C/min and nitrogen). For all materials, PCFC results showed a single peak, which occurred at the same time/temperature as a single degradation step in the corresponding thermograms, thus exhibiting a strong correlation between the two test methods.

### 3.2. Cross-Laminated Timber 

The three different layers of the orange-coloured sandwich panel CLT material were analysed in isolation with a view to identifying differences, if any, and are as follows:

- Outer squared mesh (OSM)
- Inner material (IM)
- Outer non-homogenous mesh (ONHM)

Samples from these three layers were ground into powders with a blender, to ensure high level of homogeneity. The powders were subsequently sieved through a wire-gauze grid of ca. 0.6 mm. The sieved samples were conditioned in a chamber with a relative humidity of 50%, at ca. 25 °C, for at least 48 h before further examinations. It is relevant to note here that most of the tests (as described below) were done in triplicates. 

#### 3.2.1. Thermo-gravimetric Analyses

**Figure 4:**
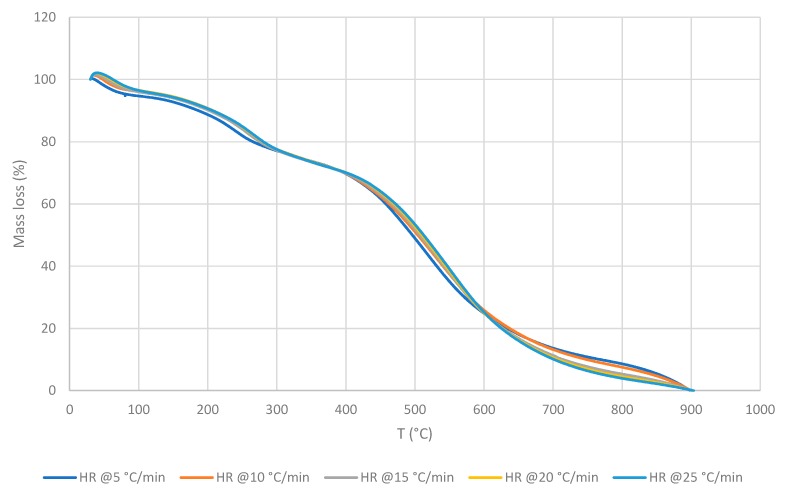
TGA traces of the outer squared mesh (OSM) layer at different HRs.

**Figure 5:**
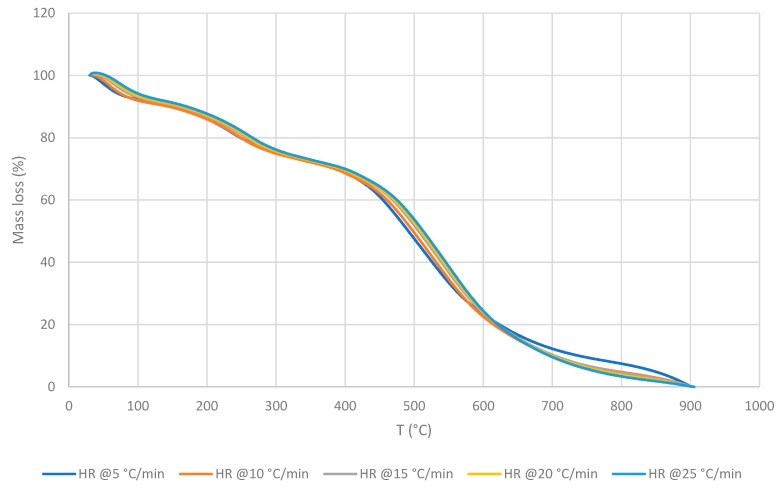
TGA traces of the inner material (IM) layer at different HRs.

**Figure 6:**
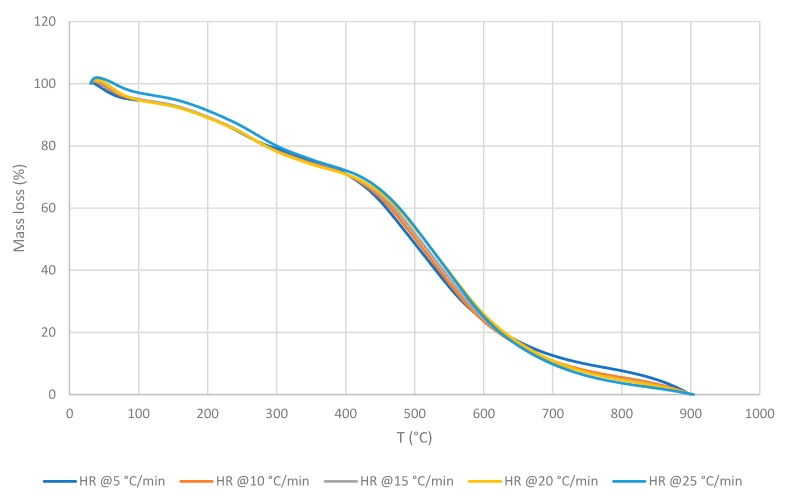
TGA traces of the outer non-homogenous mesh (ONHM) layer at different HRs.

The recorded thermograms, generally, showed two main degradation stages: The first one between ca. 100 and 300 °C, and the second stage between ca. 300 and 700 °C. The second stage appeared to be sharper and occurred over a longer temperature range compared to the first stage (Figure 4, Figure 5 and Figure 6). There is also an additional degradation phase at around 100 °C, which can be attributed to the loss of water (i.e., indicative of the moisture contents of the samples).

As can be seen from Figure 4, Figure 5 and Figure 6, the thermal degradation behaviours of the three CLT’s layers are very similar, which could indicate that their chemical constitutions are remarkably similar. Nevertheless, for the completeness of the study, all the three layers were also tested separately in the subsequent investigations. The values of the kinetic parameters of OSM and ONHM layers were found to be as follows: *E_a_* = 542.28 kJ∙mol^−1^ for the OSM at α between 0.45 and 0.65; and *E_a_* = 417.77 kJ∙mol^−1^ for the ONHM with α in the range between 0.40 and 0.70. Finally, the IM was characterised by two noticeably different stages of degradation: The first one with *E_a_* values of 173.59 kJ∙mol^−1^ at α between 0.10 and 0.22, and the second one occurring at α between 0.40 and 0.70 with associated *E_a_* values of 398.72 kJ∙mol^−1^. Here it should be noted that for the top, OSM, and the bottom, ONHM, layers, the employed MATLAB software only distinguished one main degradation step. Generally, all samples were analysed within the α range from 0.10 to 0.90. It can be clearly seen that the values of the kinetic parameters are different for the different layers. The values of *E_a_* have a direct bearing to ease the degradation of the materials under a thermal insult. Here, the OSM layer has the highest value for *E_a_* (i.e., 542.28 kJ∙mol^−1^), thus would constitute a better thermal barrier where it forms parts of a structural element. 

Generally, it was also found that the general profiles of the thermograms were quite similar, regardless of the HRs. However, the materials constituting the different layers exhibited slightly different maximum temperature values for each degradation step, and at the same time showed decreasing char yields upon the increase in the HR. The latter aspect may suggest that these variables could change with a predictable tendency if materials are exposed to the higher and the lower HR. The induction temperature values for the three layers were also strikingly similar (i.e., approximately around 135 °C).

#### 3.2.2. PCFC Studies

The PCFC results for the OSM, IM, and ONHM layers are listed in Table 4, Table 5 and Table 6. In general, the values of HRC, pHRR, THR, and *h_c_* are found to be higher for the degradation in an oxidative atmosphere compared to nitrogen. However, the temperatures to pHRR are very similar in both cases. The char yields were found to be higher in the inert stream. The only exemption was the ONHM layer with the HRC value higher in the inert atmosphere (9.0 J∙g^−1^∙K^−1^) compared to 3.7 J∙g^−1^∙K^−1^ in the oxidative atmosphere. 

Another important difference was observed for the values of the heat of combustion, *h_c_*. Usually the measured heats of combustion are almost the same under inert and oxidative streams as seen for the inner core materials of the Façade type 1 and Façade type 2 materials (see Table 2 and Table 3). However, in the case of ONHM layer, the value of *h_c_* for the oxidative stream was 65% higher than the value obtained in the inert stream—this parameter was influenced by the THR and the char yield. 

**Figure 7:**
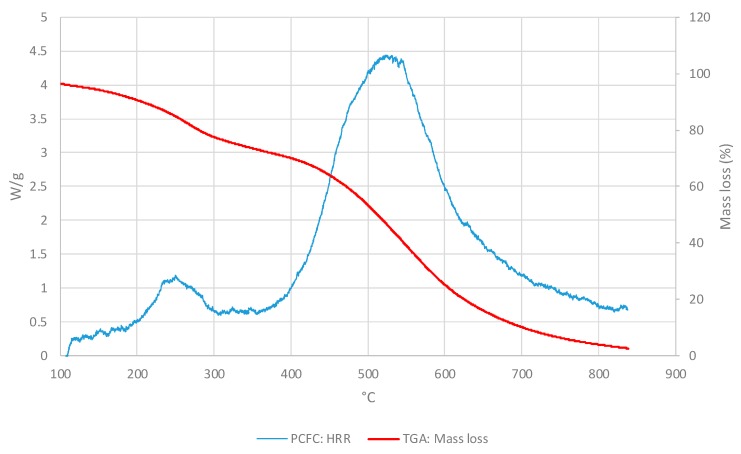
Values of HRRs (W/g) and of mass loss (wt.%) for the OSM layer.

**Figure 8:**
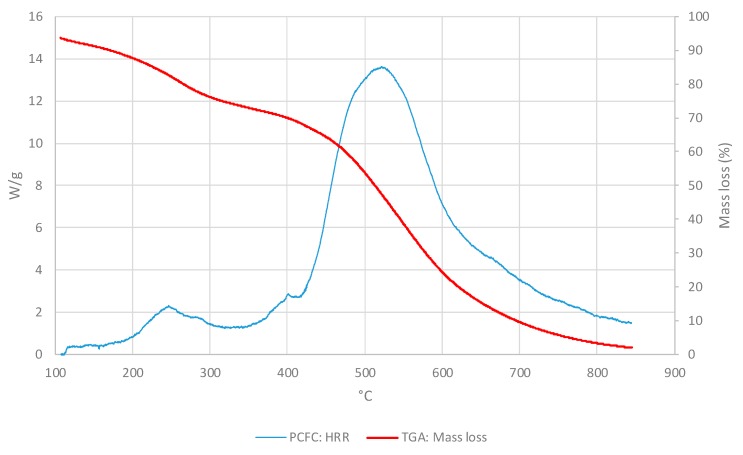
Values of the HRRs (W/g) and mass loss (wt.%) for layer IM.

**Figure 9:**
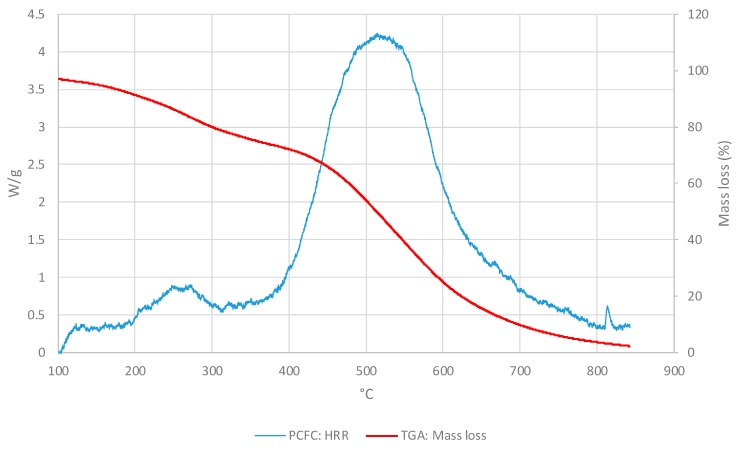
Values for HRRs (W/g) and mass loss (wt.%) for the ONHM layer.

For comparison, we have overlaid the mass loss profiles from TGA runs with the corresponding HRR curves from PCFC experiments for the three layers as given below (Figure 7, Figure 8 and Figure 9). It should be noted that both the curves were obtained in nitrogen atmosphere at the same heating rate (i.e., at 25 °C∙min^−1^ for TGA and PCFC). 

As it can be seen from the thermograms and corresponding HRR curves shown in Figure 7, Figure 8 and Figure 9, the degradation of the layers occurred in two steps. Thus, the overlays also illustrated a strong correlation between TGA and PCFC techniques. 

Moreover, when the results of PCFC (Method A) and TGA in nitrogen are compared, up to 80% differences, in some cases, regarding the char yields were observed. This clearly indicates a substantial degree of combustion inhibition at the latter stages of PCFC run, and therefore the samples can be considered more flame retarded, with the effect primarily occurring in the vapour phase. However, the maximum temperatures for degradation/combustion were found to be reasonably close. As it follows from Table 4, Table 5 and Table 6, the values of HRC, pHRR, and THR are significantly higher for the IM compared to the outer layers. This points out that the outer layers are made more fire proof than the inner core during the manufacturing process. As expected, the IM layer was found to produce the smallest amount of char residue. This can be considered as a desirable attribute of a sandwich material as the outer layers are more prone to thermal/fire exposure.

**Figure 10:**
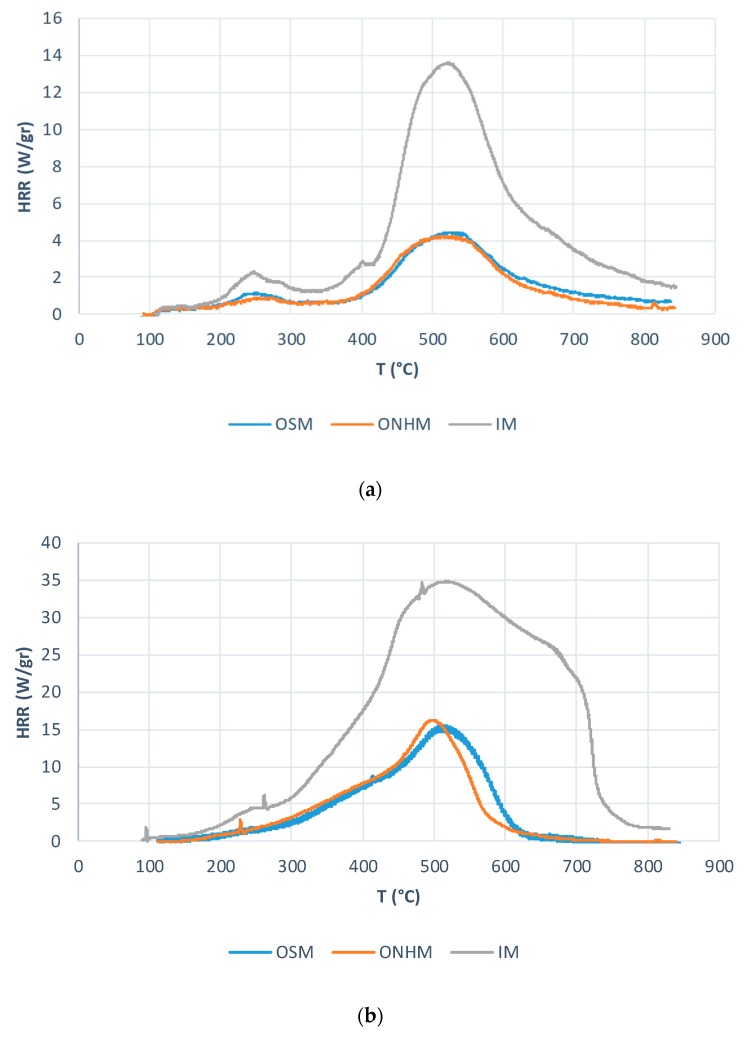
A comparison of HRR curves for the three layers in: An inert stream (**a**) and oxidative stream (**b**) of PCFC.

Finally, the values for the maximum temperature of heat release rate, $T_{max}^{*}$, and, especially heats of combustion, *h_c_*, were very close for both methods. This may point out that any preliminary indications of the performance of the different layers may not necessarily reflect how they behave in a real fire scenario. Furthermore, when we compare the HRR profiles recorded for the three layers under the two atmospheres (method A in nitrogen: Figure 10a and method B in air: Figure 10b) it is evident that the outer layers are significantly flame retarded than the inner core.

## 4. Conclusions

The results obtained in the current study provide an essential for construction sector information in relation to thermal degradation and combustion of two common materials, ACPs and CLT. The lack of such information had led to tragedies linked to fires involving tall building. Among all the materials tested in this work, the CLT demonstrated better fire-retardant attributes compared to ACPs and thus is more fire safe when used for pre-fabricated houses. In contrast, the inner core of the ACP façade materials is highly likely to contribute to the fire load/growth in a real fire scenario. This paper highlights significant fire hazards linked the use of ACPs for pre-fabricated houses around the world. The bench-scale tests revealed a drastic difference in the behaviour of different layers of ACPs. These results highlight the need of properly fire-proofing the internal core of ACPs, for example by substituting with polymers containing reactive fire retardant groups. 

The results of TGA can only be treated as a first port-of-call in gauging the flammability aspects of the materials under investigation. However, the induction temperatures, slopes of the degradative steps, and the char residues obtained from the thermograms have provided useful inferences as to the behaviours of the materials under heat/fire conditions. The PCFC runs, on the other hand, have furnished a better overall profile, especially, relating to the combustion behaviours of ACPs and CLT materials in the present study. 

It is significant to note the reasonable correlations found between the thermograms and HRR curves obtained through TGA and PFCF runs. However, a great difference in the char yields measured by the two techniques was observed. Depending on the tested material, the char yield left after the PCFC can be 4–20 times higher than the one obtained in TGA run. 

## Figures and Tables

**Table 1:** Details of the tested materials.

Material	Appearance/Morphology	Specification of the Test Specimen (Possible Chemical Nature ^1^)
ACPs—Façade type 1	Silver coloured; three-layered with a polymeric inner core; grey 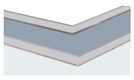	Test specimen from the internal core material (polyethylene)
ACPs—Façade type 2	Silver coloured; three-layered with a polymeric inner core; black 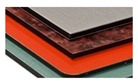	Test specimen from the internal core material (polyethylene)
CLT	Orange coloured; ca. 5 mm thick; multilayer, porous and heterogeneous in nature 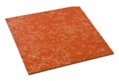	Test specimens from the upper, middle and bottom layer of CLT (ligno-cellulosic)

^1^ primarily inferred from the FT-IR spectra.

**Table 2:** Pyrolysis combustion flow calorimetry (PCFC) parameters for the Façade type 1 material.

Sample No.	Method A/Method B					
	HRC (J∙g^−1^∙K^−1^)	pHRR (W∙g^−1^)	THR (kJ∙g^−1^)	$T_{max}^{*}\;({}^{\circ}C)$	Char yield (wt.%)	*h_c_* (kJ∙kg^−1^)
1	1283.00/1886.00	528.90/780.90	37.60/34.20	478/454	9.33/4.00	41.47/35.63
2	1303.00/1835.00	544.00/686.60	37.80/35.10	479/455	8.00/6.67	41.09/37.61
3	1289.00/1298.00	520.20/612.40	36.20/35.10	478/451	6.67/5.33	38.79/37.08
Average	1291.67/1860.50	531.03/693.30	37.20/34.80	479/453	8.00/5.33	40.45/36.77

**Table 3:** PCFC parameters for the Façade type 2 material.

Sample No.	Method A/Method B					
	HRC (J∙g^−1^∙K^−1^)	pHRR (W∙g^−1^)	THR (kJ∙g^−1^)	$T_{max}^{*}\;({}^{\circ}C)$	Char yield (wt.%)	*h_c_* (kJ∙kg^−1^)
1	554.00/1210.00	231.00/492.90	17.90/18.60	465/493	60.00/62.67	44.75/49.82
2	587.00/1080.00	242.90/426.60	18.00/19.20	471/427	60.00/61.33	45.00/49.66
3	588.00/1040.00	244.40/462.00	18.30/18.60	470/462	60.00/60.00	45.75/46.50
Average	576.33/1110.00	239.43/460.50	18.07/18.80	469/380	60.00/61.33	45.17/48.66

**Table 4:** PCFC parameters for the OSM layer.

Sample No.	Method A/Method B					
	HRC (J∙g^−1^∙K^−1^)	pHRR (W∙g^−1^)	THR (kJ∙g^−1^)	$T_{max}^{*}\;({}^{\circ}C)$	Char yield (wt.%)	*h_c_* (kJ∙kg^−1^)
1	9.00/31.00	3.36/11.71	1.90/6.80	484/469	87.71/72.71	15.4/24.92
2	10.00/39.00	3.46/12.66	1.80/7.30	486/476	87.50/72.08	14.40/26.15
3	10.00/38.00	3.98/12.21	1.80/7.40	497/477	86.88/71.25	13.71/35.74
Average	9.67/36.00	3.60/12.19	1.83/7.17	489/474	87.36/72.01	14.52/25.60

**Table 5:** PCFC parameters for the IM layer.

Sample No.	Method A/Method B					
	HRC (J∙g^−1^∙K^−1^)	pHRR (W∙g^−1^)	THR (kJ∙g^−1^)	$T_{max}^{*}\;({}^{\circ}C)$	Char yield (wt.%)	*h_c_* (kJ∙kg^−1^)
1	30.00/80.00	10.57/29.83	5.60/24.07	478/459	64.50/10.25	15.77/27.52
2	30.00/81.00	10.15/30.08	5.60/24.10	479/457	64.75/9.75	15.89/26.70
3	25.00/79.00	9.18/29.04	4.50/23.40	490/453	64.75/10.00	12.77/26.00
Average	28.33/80.00	9.97/29.65	5.23/24.07	482/457	64.67/10.00	14.81/26.74

**Table 6:** PCFC parameters for the ONHM layer.

Sample No.	Method A/Method B					
	HRC (J∙g^−1^∙K^−1^)	pHRR (W∙g^−1^)	THR (kJ∙g^−1^)	$T_{max}^{*}\;({}^{\circ}C)$	Char yield (wt.%)	*h_c_* (kJ∙kg^−1^)
1	9.00/3.80	3.19/12.32	1.80/6.00	468/468	87.92/74.79	14.90/23.80
2	9.00/3.60	3.18/11.78	1.80/6.30	471/463	87.50/73.13	14.40/23.44
3	9.00/3.70	3.40/12.25	2.00/6.20	467/465	87.50/71.88	16.00/22.04
Average	9.00/3.70	3.26/12.12	1.87/6.17	469/466	87.64/73.26	15.10/23.10
